# Quality of life of women with urinary incontinence in the postpartum period: an integrative literature review

**DOI:** 10.3389/fgwh.2025.1562572

**Published:** 2025-05-01

**Authors:** Filipa Daniela Lopes, Carolina Henriques, Maria da Saudade Lopes, Isabel Margarida Mendes

**Affiliations:** ^1^ Health Sciences Research Unit, Nursing, Nursing School of Coimbra, Coimbra, Portugal; ^2^ Nursing School of Coimbra, Coimbra, Portugal; ^3^ University of Coimbra, Coimbra, Portugal; ^4^ School of Health Sciences, Polytechnic Institute of Leiria, Leiria, Portugal; ^5^ ciTechCare—Center for Innovative Care and Health Technology, Polytechnic Institute of Leiria, Leiria, Portugal

**Keywords:** women, urinary incontinence, postpartum period, quality of life, nursing

## Abstract

Urinary incontinence (UI) during pregnancy and after childbirth can negatively impact women's lifestyles, health, and well-being. It is a common problem that is often normalized by both healthcare professionals and women. This integrative review examines the influence of UI on women's quality of life (QoL) during the postpartum period and identifies the main affected domains. It will also contribute to the discussion of the results of a primary study. Fourteen studies were included in this review, mainly from Brazil and Spain, with most using quantitative methods. The findings indicate that UI negatively affects the QoL of women during the postpartum period, with mixed UI causing greater discomfort despite stress UI being more frequent. The main affected domains are general health perception, physical functioning, daily life activities, psychological/emotional/mental and social aspects, and sexuality. Effective interventions should promote women's self-care and enhance their awareness and recognition of the problem. These interventions should go beyond physical aspects to address emotional and psychological dimensions, such as low self-esteem and self-image, secrecy, embarrassment, and reluctance to seek help. Nurse midwives have specialized skills and can work within multidisciplinary teams to improve the QoL of women with UI at a time of increased vulnerability.

## 1 Introduction

Urinary incontinence (UI) during pregnancy and after childbirth is a common problem that is often normalized by both healthcare professionals and women, which can lead to changes that negatively impact women's lifestyles, health, and well-being in the postpartum period (1).

Postpartum UI is defined as the involuntary loss of urine experienced during the postpartum period and up to 12 months after delivery. Among its types, postpartum stress urinary incontinence (SUI) is the most common and is characterized by involuntary loss of urine during physical effort, exertion, sneezing, or coughing (2). Postpartum urgency urinary incontinence (UUI) refers to the involuntary loss of urine associated with a sudden and urgent need to urinate. Lastly, postpartum mixed urinary incontinence (MUI) is defined as the involuntary loss of urine associated with both urgency and physical effort, exertion, sneezing, or coughing (2).

Between six weeks and one year postpartum, the weighted mean prevalence of UI is 31%, ranging from 10% to 63%. A decrease in prevalence is typically observed during the first three months postpartum due to the natural recovery of the pelvic floor, followed by an increase associated with the return to daily activities (3).

Pregnancy and childbirth are risk factors for the occurrence of postpartum UI (4, 5), especially postpartum SUI (1, 4, 6). Women who experience urine leakage during pregnancy are more likely to develop postpartum UI (4, 7). Other factors include pre-pregnancy changes, physiological changes related to pregnancy, and lack of guidance from healthcare professionals (5).

Postpartum UI is often underestimated and perceived as a normal consequence of childbirth by both healthcare professionals and women (4). This perception leads to a lack of effective preventive interventions and low rates of help-seeking, diagnosis, or treatment among women (8).

Quality of life (QoL) is a subjective concept that can be difficult to discuss scientifically due to its widespread use in everyday language (9). The World Health Organization defines QoL as an individual's perception of their position in life in the context of their culture and value systems and in relation to their goals, expectations, standards, and concerns. It encompasses their physical health, psychological state, level of independence, social relationships, personal beliefs, and relationship with the environment. Measuring QoL helps assess the impact of environment, disease, and health interventions (10).

QoL assessment instruments have been developed across different cultural contexts and usually include six domains: physical, psychological, level of independence, social relationships, environment, and spirituality/religion/personal beliefs (10).

However, addressing QoL during the postpartum period—a phase that profoundly impacts all its domains—requires recognizing it as a time of increased vulnerability for women. This period is marked by significant physiological, psychological, and social changes, as women adapt to a new reality, identity, and role (11). In this process, women should understand that motherhood is a role that does not exclude them from other areas and that self-care should not take second place to caregiving duties.

This integrative literature review aims to explore the influence of UI on women's QoL during the postpartum period and identify the main affected domains. It serves as the starting point for a future primary study that will assess the QoL of women with postpartum UI within the first 12 months after childbirth. In parallel, a phenomenological primary study will be conducted to explore the lived experiences of these women. By synthesizing the findings from these studies, this integrative literature review contributes to a deeper understanding of the topic.

The PCC (Population, Concept, and Context) mnemonic (12) was used to outline the following review questions: Q1: How does UI influence women's QoL during the postpartum period? and Q2: What are the main domains of women's QoL affected by UI during the postpartum period?

A preliminary search was conducted to identify recent studies addressing these questions. While there are reviews on QoL in the postpartum period, many do not specifically address UI or examine the QoL of women with UI without specifically focusing on the postpartum period. Some reviews explore risk factors (13), prevalence and treatment options (14), or the impact of UI on the QoL of postpartum women but exclude multiparous women and those over 40 years old (15).

## 2 Materials and methods

The integrative review method was used to achieve the objective of this review. This method combines research studies conducted using different methodologies and synthesizes knowledge to better understand the current state of evidence on a specific phenomenon. It is particularly valuable to understand issues relevant to health care and public policies (16). The integrative review method consists of five stages: problem identification, literature search, data evaluation, data analysis, and data presentation (16).

A literature search was conducted between July and October 2024, covering articles published between 1997 and 2024. This time frame was chosen because the King's Health Questionnaire (KHQ), one of the first specific instruments to assess QoL in women with UI, was published in 1997 (17). Unlike broader QoL assessment tools, the KHQ is tailored specifically to UI. Currently, there is no specific instrument to assess postpartum UI, nor has a systematic literature review on this specific topic been published after this date.

**Figure 1:**
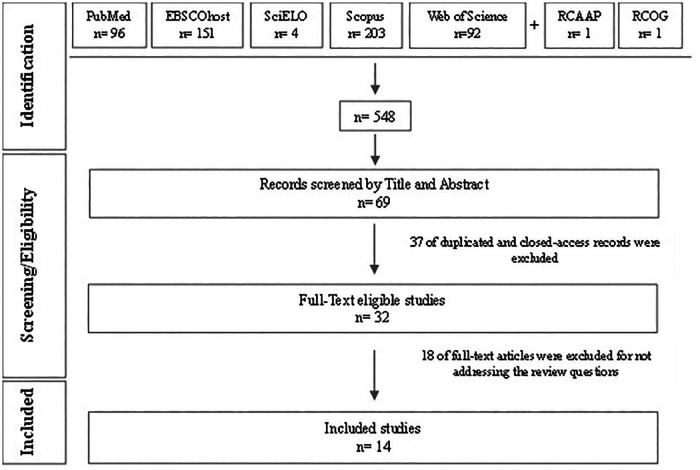
Flowchart of the study selection process adapted from the PRISMA-ScR flowchart (46).

This review includes studies that focused on women with postpartum UI up to one year after childbirth and that address QoL assessment, regardless of the type of study or data collection instrument. Full-text articles available in Portuguese, English, Spanish, and French were considered eligible. The literature search was conducted in multiple databases: MEDLINE (via PubMed), CINAHL Complete (via EBSCO), Nursing & Allied Health Collection: Comprehensive (via EBSCO), MedicLatina (via EBSCO), Academic Search Complete (via EBSCO), SciELO, Web of Science, Scopus, and the Open Access Scientific Repositories of Portugal (RCAAP). Additionally, specific guidelines, such as those from the Royal College of Obstetricians and Gynaecologists (RCOG), were consulted. The search query used was: “quality of life AND urinary incontinence AND postpartum”. The query was adapted to each search engine and inclusion criteria were applied. Two independent reviewers screened the studies. There was no need for a third reviewer to resolve any discrepancies. After title/abstract and full-text screening, 14 studies published between 2004 and 2023 were included in this review based on their relevance and the inclusion criteria (Figure 1).

## 3 Results

The included studies were published between 2004 and 2023, with three published in the last five years (18–20). Most of the studies were from Brazil and Spain and used quantitative methods. While most studies focused on postpartum women, three studies included participants during pregnancy (21–23). The most used instruments to assess QoL were the KHQ, the 36-Item Short Form Survey (SF-36), and the Incontinence Impact Questionnaire, Short Form (IIQ-7). The KHQ and the IIQ-7 are instruments related to the health-related QoL (HRQoL) of women with UI (17) and the impact of UI on women's lives (24), respectively.

Data were extracted using a table that included the following elements: author(s)/year of publication/country, type of study/methodology, population/sample, data collection instrument, and main findings (Table 1) to facilitate data analysis and presentation.

**Table 1:** Summary of the studies included in the review (*n* = 14).

Author Year Country	Type of study Methodology	Population/sample	Data collection instrument	Main findings
Lin et al. 2018 Taiwan (21)	Quantitative Observational, longitudinal cohort study	Pregnant and postpartum women up to 12 months after childbirth Sample: *N* = 866	IIQ-7	• More than half of the women experienced SUI during pregnancy and most of them recovered quickly in the first year postpartum; • Both SUI during pregnancy and postpartum showed a negative impact on the QoL of women who had a vaginal delivery.
Lopes 2014 Brazil (28)	Quantitative Observational, prospective cohort study	Postpartum women Sample: *N* = 358	KHQ	• UI negatively impacts HRQoL; • Women reported lower HRQoL levels in the domains of general health perception, severity measures, incontinence impact, daily activity limitations, and physical limitations.
Lima and Lopes 2016 Brazil (25)	Quantitative Observational, correlational, cross-sectional study	Postpartum women with UI Sample: *N* = 220	KHQ	• Urine leakage during sexual intercourse, though infrequent, disrupted sexual activity; • Higher mean scores were observed in the domains of general health perception, incontinence impact, and severity measures.
Liang et al. 2021 China (18)	Quantitative Observational, cross-sectional cohort study	Primiparous women between 6 and 8 weeks postpartum Sample: *N* = 130	I-QoL IIQ-7	• The greater the symptom severity, the stronger the impact on QoL among women with postpartum UI. • The decline in scores across the three dimensions of QoL (behavioral impact, psychological impact, and social activities impact) had overlapping effects; • The coexistence of SUI and UUI is an uncomfortable condition for women in the postpartum period and can significantly affect their QoL.
Arrue et al. 2010 Spain (33)	Quantitative Observational, prospective study	Primiparous women with vaginal delivery and postpartum SUI Sample: *N* = 396	ICIQ-UI-SF	• 15.1% of women reported experiencing postpartum SUI, with most cases classified as mild or moderate; • The overall impact on QoL was low.
Handa et al. 2007 USA (30)	Quantitative Longitudinal study	Postpartum women 6 months after delivery Sample: *N* = 759	SF-12	• UI had a significant impact on women's QoL, negatively affecting social, economic, psychological, and sexual aspects.
Leroy and Moraes Lopes 2012 Brazil (31)	Quantitative Observational, case-control study	Postpartum women 90 days after delivery Sample: *N* = 344	ICIQ-SF SF-36 KHQ	• Postpartum UI, although small in quantity, is frequent and significantly impairs daily life and QoL; • High impact of UI in the domains of incontinence impact, emotions, daily activity limitations, and physical limitations; • HRQoL of the continent and incontinent postpartum women differed across several domains, including physical aspects, pain, general health status, vitality, social aspects, and mental health, with incontinent women experiencing worse outcomes; • UI significantly affects the physical and mental health of postpartum women, especially those with MUI, with a greater impact on HRQoL.
Martínez-Galiano et al. 2019 Spain (19)	Quantitative Cross-sectional cohort study	Women who gave birth in the year 2017 in Spain Sample: *N* = 2,990	SF-36	• The primary factors leading to a decline in QoL were depressive symptoms, lactation problems, difficulties in sexual relations after childbirth, and UI;
				• UI affects three domains: physical, psychological, and overall health.
Li et al. 2023 China (20)	Qualitative Exploratory study with thematic analysis	Postpartum women (6 weeks to 1 year) who underwent pelvic physiotherapy for SUI Sample: *N* = 22	Semi-structured interviews	• Many women reported dissatisfaction with their QoL as one of the motivators for seeking PFPT; • SUI had a negative impact on women's overall well-being and interfered with their daily activities and social lives.
Walton et al. 2014 USA (26)	Quantitative Prospective, cross-sectional, and correlational study	Bangladeshi women with a history of one or more obstetric deliveries in the past three years Sample: *N* = 94	SF-36 IIQ-7	• The presence of UI showed a strong inverse correlation with HRQoL; • The high incidence of UI through the third year postpartum indicates that UI does not resolve itself automatically and requires further interventions; • Women who reported UI scored significantly lower in the physical, pain, general health, and emotional domains than women who did not report UI.
Van der Woude et al. 2014 Netherlands (29)	Systematic literature review	Studies with samples consisting of postpartum women Sample: *N* = 66 studies	The most frequently used: SF-36 WHOQOL-Bref IIQ-7 IIQ	• All three domains of QoL, physical, psychological, and social, were impaired in postpartum women with UI, with even worse QoL in women with MUI; • The presence of UI, especially MUI, can negatively impact the physical QoL of postpartum women; • Women with MUI have worse mental QoL scores than those with only SUI or UUI. • Women with MUI tend to have worse social QoL scores compared to those with only SUI or UUI; • In postpartum women, QoL impairment seems to be associated with UI, and health status impairment appears to be associated with postpartum depression and cesarean sections.
Triviño-Juárez et al. 2017 Spain (27)	Quantitative Prospective cohort study	Primiparous postpartum women Sample: *N* = 546	SF-36	• At the sixth week postpartum, women with postpartum UI reported a lower HRQoL; • Between the sixth week and sixth month postpartum, HRQoL improved for all modes of birth; • Women who reported the lowest HRQoL were those with postpartum UI. Most women with postpartum UI were in the forceps-assisted delivery group.
Valeton and Amaral 2011 Brazil (22)	Quantitative Prospective cohort study	Women in the third trimester of pregnancy and in the postpartum period Sample: *N* = 343; *N* = 236	KHQ	• UI significantly impacted QoL, despite an improvement in QoL during the postpartum period.
Dolan et al. 2004 UK (23)	Quantitative Prospective cohort study	Primigravidae women between 34 and 40 weeks Sample: *N* = 492	KHQ	• QoL was worse in women with postnatal UI, compared to those with prenatal incontinence: • The impact of UI on QoL was greater in the postnatal period, especially in women with other lower urinary tract symptoms or MUI or UUI; • Women who experience UI show a deterioration in general and personal health in the postpartum period; • UI and health status impairment seem to be associated with postpartum depression and cesarean sections.

HRQoL, health-related quality of life; ICIQ-UI-SF, International Consultation on Incontinence Questionnaire-Urinary Incontinence-Short Form; IIQ-7, incontinence impact questionnaire-7; I-QoL, incontinence quality of life instrument; KHQ, king's health questionnaire; MUI, mixed urinary incontinence; QoL, quality of life; SF-12, 12-item health survey; SUI, stress urinary incontinence; UI, urinary incontinence; UUI, urgency urinary incontinence; ICIQ-SF, International Consultation on Incontinence Questionnaire-Short Form; PFPT, pelvic floor physical therapy; SF-36, study 36—item short form health survey; IIQ, incontinence impact questionnaire; WHOQOL-Bref, World Health Organization Quality of Life Assessment-Bref.

### 3.1 Q1—how does UI influence women’s QoL during the postpartum period?

UI has a negative impact on women's QoL during pregnancy and after childbirth, persisting at least until 12 months postpartum, especially SUI (20, 25).

The impact of UI on QoL is greater during the postpartum period than during pregnancy due to lifestyle changes after childbirth or post-partum related morbidities (22).

UI is strongly associated with lower with HRQoL (26, 27). Among the different types of UI, MUI has the greatest negative impact, causing more discomfort than SUI and UUI (18, 28, 29), even though SUI is the most common type (25).

At six months postpartum, UI continues to have a significant impact on women's QoL (30). However, the same study found no correlation between the type of UI and QoL.

UI is one of the leading factors contributing to a decrease in QoL, alongside other issues such as depressive symptoms, lactation problems, and postpartum sexual health concerns (19).

Although its prevalence is high in the postpartum period, UI is even more common during pregnancy, especially in the third trimester (25). It occurs more frequently after vaginal delivery (21), with most women with postpartum UI having had a forceps-assisted delivery (23, 27). The incidence of UI reaches 45% between one and three years postpartum (26).

Although UI is usually mild to moderate, cases of severe UI also occur (18, 25). Even when the volume of leakage is small, its frequent occurrence can significantly disrupt daily life and reduce QoL during the postpartum period (31). As postpartum UI symptoms become more severe, their impact on women's QoL increases (18), often resulting in lifestyle changes such as using absorbent pads to avoid wetting clothes and limiting physical and social activities to avoid potentially embarrassing situations. However, these restrictive behaviors may lead to psychological discomfort and negatively affect both physical health and interpersonal relationships (18). Urine leakage during sexual activity also has a negative impact on women's sexual lives, with most partners being aware of their UI (25). In addition, urine leakage during coughing, sneezing, or physical activities is common (31).

### 3.2 Q2—what are the main domains of women’s QoL affected by UI during the postpartum period?

The included studies highlight some of the most affected QoL domains due to postpartum UI. These domains were grouped according to the WHOQOL Group's definition of QoL (10) and the terminology used by the respective authors.

Women with UI scored lower in the following domains: general health perception (19, 23, 25, 26, 28, 31), physical limitations (19, 26, 28, 29, 31), daily life activities (20, 28, 31), psychological/emotional/mental aspects (18, 19, 26, 29–31), social aspects (18, 20, 22, 29–31), and sexuality (25).

Other domains include incontinence impact (25, 28, 31), behavioral impact (18), economic impact (30), sleep and energy (22, 23), and vitality (31).

## 4 Discussion

The analysis of the included studies reveals a predominance of research from Brazil and Spain, with most studies focusing on postpartum women. In contrast, only a few studies examined pregnancy-related issues. Although postpartum UI is typically defined as the period from childbirth up to one year postpartum (2), both pregnancy and childbirth are considered risk factors for UI. Moreover, pregnant women experiencing urine leakage are more likely to develop postpartum UI (4, 7). Therefore, some studies addressing postpartum UI include data from the pregnancy period (21–23).

Although the research includes studies published up to and including 2024, only three were published in the last five years. Notably, the majority of these studies used quantitative methods. This predominance of quantitative research underscores the need for qualitative studies, which could provide deeper insights into how and why postpartum UI affects key QoL domains.

One of the most used instruments to assess QoL was the KHQ. The KHQ has proven reliable and valid for evaluating the quality of life in women with UI. It considers various dimensions, including personal and daily life limitations, emotional aspects, social relationships, and urinary symptoms. Its comprehensive approach is valuable for guiding effective interventions and ensuring appropriate clinical follow-up and treatment (32).

This review highlighted the importance of recognizing UI as a frequent problem during the postpartum period and its significant impact on the QoL of postpartum women.

Among studies assessing the prevalence or incidence of UI, SUI emerges as the most frequent type following childbirth (21, 25, 31), although one study suggests that MUI is more prevalent (23). A systematic review on the prevalence and incidence of UI further confirms that SUI is the most prevalent type during the postpartum period (3). Nevertheless, women with MUI generally report a lower QoL than those with SUI, particularly in terms of restrictive behavior. This is attributed to the combined symptoms of SUI and UUI, with MUI causing greater discomfort (18, 28). Even the study reporting a low impact of UI on QoL acknowledges the presence of moderate to mild symptoms six months postpartum (33).

The majority of the selected studies highlight the limitations imposed by postpartum UI on physical functioning, daily life activities, and social domains (18–20, 22, 26, 28–31). These findings support the conclusions of a 2022 literature review on the impact of UI on QoL among postpartum women (15), which noted a tendency toward isolation. In addition, these factors can also negatively affect emotional and psychological well-being, potentially contributing to depressive symptoms (15), as suggested by some of the selected studies (18, 19, 26, 29–31). While UI increases depressive moods in postpartum women, its impact extends further, affecting their emotional well-being, emotional reactions, energy levels, sleep, physical activities, and social relationships (34). Furthermore, UI negatively affects their clothing choices, participation in leisure activities, daily routines, QoL, and overall health (35). A systematic review on the association between UI and postpartum depression found that UI increases the risk of developing postpartum depression by 45% (36).

The stigma surrounding postpartum UI arises from widespread misconceptions that view it as a deviation from the norm, often linked to poor hygiene and lack of self-control. These perceptions contribute to self-stigma, which undermines self-esteem, increases anxiety, and discourages women from seeking healthcare (37). Rooted in community values associating UI with uncleanliness, this stigma is often internalized, worsening mental health. Although some women are aware of the negative impact of UI on their mental health, many still feel embarrassed to discuss their condition with friends and family (37).

One of the selected studies—a theoretical-critical reflection on women's knowledge of UI (35)—highlights that fear of exposing their feelings, dignity, and self-worth to both professionals and society often compels women to conceal their condition. This concealment can compromise bodily autonomy, affecting partner acceptance, sexuality, self-image, and self-esteem. As a result, many women avoid seeking help or engaging with prevention, treatment, and recovery efforts for UI. A study on help-seeking behavior among postpartum women with UI in China and Indonesia found that, despite the high prevalence of UI, only half of the affected women sought professional help. Barriers to seeking help included insufficient social support, lack of awareness regarding available treatment options, and a lack of proactive engagement from healthcare professionals in addressing UI during consultations. Additionally, factors such as symptom severity and perceived QoL were found to negatively influence the likelihood of seeking professional help (38).

Dissatisfaction with QoL is considered a key motivator for seeking treatment, particularly pelvic floor physical therapy (20). To overcome barriers to help-seeking behavior, it is essential to improve access to healthcare services, strengthen social support systems, and ensure that women have easy access to healthcare professionals who can provide accurate information and appropriate treatment options (38). Similarly, a study examining barriers to help-seeking behavior among women with stigmatized pelvic health symptoms, including UI, identified stigma, lack of knowledge, and the minimization of symptoms by healthcare professionals as significant obstacles. This study recommend strategies to reduce stigma, increase symptom awareness, provide training for healthcare professionals, and improve access to healthcare services as key enablers of help-seeking behavior (39).

Although addressed by only one of the selected studies, specifically one that assesses the extent to which UI affects QoL (25), without controversy from the others, the impact of UI on sexual activity and partner acceptance is evident, with sexuality identified as one of the QoL domains affected by UI. A study on factors associated with persistent sexual dysfunction and pain 12 months postpartum found that the continued presence of bothersome postpartum UI, along with higher levels of patient-perceived daily stress, was strongly associated with sexual dysfunction (40).

All affected QoL domains, particularly the decline in general health perception, are closely linked not only to childbirth and postpartum morbidities but also to lifestyle changes and the adjustments required after a child's birth. Four of the selected studies specifically address the impact of UI on HRQoL (23, 26, 28, 31). During the postpartum period, women tend to prioritize their baby's needs over their health, delaying self-recognition of symptoms and help-seeking behaviors aimed at improving QoL (34).

The normalization of UI by healthcare professionals is highlighted in a study on the main factors contributing to the development of UI in women during the postpartum period (5), as well as in a study examining risk factors for postpartum UI among nulliparous women (8). It is recommended that healthcare professionals recognize UI as a prevalent condition that affects the QoL of postpartum women, as this recognition could facilitate women's self-recognition of their symptoms and break the cycle of omission, neglect, or complacency from both patients and professionals. This need for awareness is further emphasized in a study aimed at sensitizing healthcare professionals and health managers to the scope and implications of UI-related issues (35).

The importance of qualified, multidisciplinary care in preventing, treating, and guiding women who may dismiss their symptoms as irrelevant (15) during the postpartum period cannot be underestimated (22).

Although the selected studies do not focus primarily on solutions, all address the need for intervention in their conclusions, as UI is a condition that does not resolve spontaneously and impacts multiple domains of QoL. The recommended interventions include early screening for risk factors (21, 22, 25, 28, 29), educational interventions (18, 23, 25, 26, 28, 31), emotional and psychological support (19, 20, 23, 25, 27, 29, 30), and pelvic floor rehabilitation programs (18, 20, 22, 25, 27, 30, 31, 33).

Patient perspectives are essential for improving clinical care, as they emphasize the importance of listening to patient concerns and optimizing treatments to better meet their needs (41). Effective two-way communication between clinicians and patients facilitates shared decision-making (20). Given the significant impact of UI on QoL, clinicians caring for postpartum women should routinely screen for UI and provide appropriate evaluation and treatment options (30). Increased awareness and attention from healthcare professionals, particularly nurse midwives, can support the development of effective health interventions. These professionals play a crucial role in the prevention, guidance, and management of health issues affecting women throughout their life cycle, including the prenatal and postpartum periods.

Enhancing patient education and equipping healthcare professionals with the skills to address UI sensitively and provide appropriate treatment are essential. Implementing standardized assessments and stigma-reducing strategies, such as supervised physical activity programs and the creation of safe, inclusive spaces, can significantly improve outcomes for women with postpartum UI (42).

In addition, the National Institute for Health and Care Excellence (*N*ICE) guidelines emphasize the importance of cultural sensitivity when addressing pelvic floor dysfunction. Many women may feel embarrassed discussing their symptoms and may assume that healthcare professionals are equally uncomfortable with the topic. To provide more effective support, the use of digital information sources, such as apps or videos, and a community-based multidisciplinary team approach is recommended. Encouraging lifestyle changes and providing a supervised pelvic floor muscle training program for at least three months are essential. Additionally, the psychological impact of symptoms should be discussed and integrated into a management plan (43).

Lifestyle changes include weight loss (since excess weight is a risk factor), timed voiding to prevent excessive bladder filling (particularly in cases of SUI), and fluid restriction (44). Pelvic floor physical therapy is considered an effective first-line treatment, either alone or combined with biofeedback, electrostimulation, or the use of vaginal cones, for reducing UI and improving pelvic floor muscle contraction (44, 45). Pharmacotherapy is commonly used in the treatment of UUI, with antimuscarinic agents (which inhibit involuntary detrusor muscle contractions) and beta-agonists (which relax the detrusor muscle and increase bladder capacity) being the main classes of medication. Vaginal estrogen may also be considered, particularly for postmenopausal women. In more severe cases, surgery, such as the placement of a synthetic sling to support the bladder neck, may be an option (44).

It is crucial to reflect on the role of healthcare professionals and highlight the importance of multidisciplinary and intersectoral collaboration in healthcare, ultimately improving the QoL of women with UI during the postpartum period, which is particularly vulnerable.

This review has some limitations, including the restriction to articles published in Portuguese, English, Spanish, and French, which may have led to the exclusion of relevant studies in other languages. Additionally, the heterogeneity of the included studies—with varying sample characteristics and measurement instruments—may affect the generalizability of the findings.

## 5 Conclusion

This integrative review included studies published on various databases, as well as gray literature, over an extended time period, reflecting the lack of reviews addressing the proposed objective.

UI is a common condition that often goes unrecognized by both postpartum women and healthcare professionals. It has a negative impact on the QoL of women during the highly vulnerable postpartum period. The most affected QoL domains include general health perception, physical limitations, daily life activities, psychological/emotional/mental and social aspects, and sexuality.

Despite its high prevalence, UI should not be normalized or overlooked by healthcare professionals. Recognizing and valuing women's perceptions of their health and QoL is essential for increasing awareness of the problem among both women and healthcare professionals. This, in turn, can drive the development and integration of effective strategies in education and clinical practice.
